# *Anaplasma phagocytophilum* AFAP targets the host nucleolus and inhibits induced apoptosis

**DOI:** 10.3389/fmicb.2024.1533640

**Published:** 2025-01-07

**Authors:** Daxiu Zhang, Lifeng Yu, Hui Tang, Hua Niu

**Affiliations:** ^1^Clinical Laboratory Center, Affiliated Hospital of Guilin Medical University, Guilin, Guangxi, China; ^2^Laboratory of Hepatobiliary and Pancreatic Surgery, Affiliated Hospital of Guilin Medical University, Guilin, Guangxi, China; ^3^Guangxi Key Laboratory of Molecular Medicine in Liver Injury and Repair, Guilin Medical University, Guilin, Guangxi, China; ^4^Guangxi Health Commission Key Laboratory of Basic Research in Sphingolipid Metabolism Related Diseases, Affiliated Hospital of Guilin Medical University, Guilin, China

**Keywords:** *Anaplasma phagocytophilum*, type IV secretion system, proteomic analysis, apoptosis, nucleolus

## Abstract

*Anaplasma phagocytophilum*, the etiologic agent of human granulocytic anaplasmosis (HGA), is an obligate intracellular Gram-negative bacterium. During infection, *A. phagocytophilum* transfers its type IV secretion system (T4SS) effector proteins into host cells to manipulate cellular processes. AFAP (an actin filament-associated *Anaplasma phagocytophilum* protein) was identified as a T4SS effector protein and found to interact with the host nucleolin, as described in a previous study. In this study, proteomic analysis was performed to extensively identify AFAP-interacting proteins in host cells and analyze the potential role of AFAP in modulating host cellular processes. A total of 586 host proteins were identified interacting with AFAP by data-independent acquisition mass spectrometry and annotated to 501 Gene Ontology (GO) terms, with the significantly over-represented ones related to ribosomes, nucleolus, DNA binding, and rRNA metabolic process. Given the role of the nucleolus in cellular stress response, the targeting of AFAP to the nucleolus, and the identification of dozens of AFAP-interacting proteins that were annotated to the GO term (GO:0072331, signal transduction by p53 class mediator), the role of AFAP in modulating host apoptosis was determined. AFAP was found capable of inhibiting induced apoptosis. Thus, the proteomic analysis of AFAP-interacting proteins and determination of AFAP with anti-apoptotic activity may help elucidate the role of this T4SS effector protein in HGA pathogenesis.

## 1 Introduction

*Anaplasma phagocytophilum*, a Gram-negative obligatory intracellular bacterium (rickettsia), causes the emerging tick-borne zoonosis called human granulocytic anaplasmosis (HGA), which is characterized by fever, malaise, headache, myalgia, arthralgia, leukopenia, thrombocytopenia, and elevations in serum hepatic aminotransferases (Bakken and Dumler, 2015). During infection, multiple *A. phagocytophilum* T4SS effector proteins are delivered into host cells to modulate cellular processes by targeting different subcellular organelles or components (Rikihisa, 2017). Secreted ankyrin repeat domain-containing protein A (AnkA) targets two distinct host subcellular locations: one is the cytoplasm, where AnkA binds Abl-interactor 1 (Abi-1) to form a complex with Abl-1, stimulating Abl-1 kinase activity, or binds the Src homology 2 (SH2) domains of the non-receptor tyrosine phosphatase Src homology protein (SHP)-1, dephosphorylating phosphoproteins (Jw et al., 2007; Lin et al., 2007) and the other is the nucleus, where AnkA binds DNA to epigenetically modify chromatin structure and transcriptional programs (Dumler et al., 2016). *Anaplasma* translocated substrate 1 (Ats-1) targets mitochondria and endoplasmic reticulum (ER) to inhibit apoptosis and initiate autophagosome formation, respectively (Niu et al., 2010; Niu et al., 2012). HGE-14 (APH_0455) was found to target the host nucleus and dampen the production of reactive oxygen species (Sinclair et al., 2015). *Anaplasma phagocytophilum* tick effector A (AteA) localizes with cortical actin filaments and is critical for *A. phagocytophilum* survival in tick cells (Park et al., 2023). ER-Golgi exit site protein of *Anaplasma* (EgeA) targets ER by binding TANGO1 and SCFD1 to reduce cargo congestion at ER-Golgi exit sites (Wang et al., 2024).

The nucleolus is a membraneless subnuclear compartment that is primarily responsible for ribosomal RNA transcription and processing and ribosome assembly. Proteomic analysis revealed over 4,500 nucleolus-localized proteins, with only 30% associated with ribosome biogenesis. The remaining proteins are involved in numerous cellular processes, including cell stress sensing, cell cycle control, DNA replication and repair, and apoptosis (Yang et al., 2018). As these cellular processes are important for the replication of intracellular bacteria, nucleolar proteins are targeted to modulate their locations and activities through the bacterial effector proteins. For instance, *Brucella abortus* effector proteins NyxA and NyxB target the host nucleolar protein SENP3 (sentrin-specific protease 3), causing the mislocalization of nucleolar proteins and promoting intracellular replication of this pathogen (Louche et al., 2023); *Vibrio parahaemolyticus* delivers a T3SS effector protein, VgpA, into the host nucleolus in which it binds Epstein–Barr virus nuclear antigen 1-binding protein 2 (EBP2), recruiting c-Myc and stimulating host cell growth (Hu et al., 2021); *Coxiella burnetii* effector protein NopA (nucleolar protein A) targets small GTPase Ran (Ras-related nuclear protein), causing the nucleolar accumulation of Ran-GTP and perturbing the nuclear import of transcription factors of the innate immune signaling pathway (Burette et al., 2020); *Legionella pneumophila* effector protein LegAS4 targets heterochromatin protein 1 in the host nucleoli to activate rDNA transcription (Li et al., 2013); and Enteropathogenic *Escherichia coli* effector protein EspF targets nucleolus and mobilizes nucleolin and other proteins from nucleolus to cytoplasm (Dean et al., 2010).

In the previous study, we identified host nucleolin as AFAP (actin filament-associated *Anaplasma phagocytophilum* protein)-interacting protein and found that AFAP was colocalized with nucleolin at the cell periphery, enhancing cell adhesion (Tang et al., 2023). Nucleolin is a multifunctional protein that participates in a variety of cellular processes such as ribosome biogenesis, transcriptional regulation, cell proliferation, and apoptosis (Ginisty et al., 1999). Nucleolin is mainly found in the nucleus as a major component of the cell nucleolus. The proteomic analysis showed that 144 proteins are associated with nucleolin and involved in ribosome biogenesis, pre-mRNA metabolism, and other cellular processes (Salvetti et al., 2016). In this study, we found that AFAP targets the host nucleolus, interacts with 586 proteins determined using the proteomic analysis, and participates in numerous cellular processes based on the Gene Ontology (GO) enrichment analysis, including ribosome biogenesis and signal transduction by p53 class mediator. The effect of AFAP on one of the biological processes, signal transduction by p53 class mediator, was determined, and it was found that AFAP was capable of inhibiting induced apoptosis. Thus, the proteomic analysis of AFAP-interacting proteins, the GO term enrichment analysis of these proteins, and the determination of AFAP with anti-apoptotic activity may help elucidate the role of AFAP in HGA pathogenesis.

## 2 Materials and methods

### 2.1 Cell cultures and proteomic analysis

Human embryonic kidney 293 cells (HEK293) and monkey endothelial cell line RF/6A cells were propagated at 37°C and 5% CO2 in Dulbecco’s modified Eagle’s medium (DMEM) (Gibco) and ATCC modified MEM, respectively, supplemented with 10% fetal bovine serum (BBI Life Sciences, Shanghai, China). For the proteomic analysis, HEK293 cells stably transfected with plasmid pTAP, which expresses streptavidin-binding peptide-3xFLAG tag (SF), or plasmid pTAP-AFAP, which expresses AFAP-streptavidin-binding peptide-3xFLAG tag fusion protein (AFAP-SF), were subjected to tandem affinity purification to isolate AFAP-interacting proteins, as described previously (Tang et al., 2023), followed by data-independent acquisition mass spectrometry (DIA-MS), which was commercially performed by Applied Protein Technology, Shanghai, China. Briefly, HEK293 cells propagated in 150 mm^2^ cell culture dishes were lysed on ice with lysis buffer (30 mM Tris–HCl, 150 mM NaCl, and 0.5% (v/v) Nonidet-P40, pH 7.4), supplemented with protease inhibitor cocktail and phosphatase inhibitors (APExBIO). After clearance by centrifugation at 12000 *g* for 10 min at 4°C, the supernatants were incubated with streptavidin resin (GenScript, Nanjing, China) for 2 h at 4°C. After washing four times with washing buffer (30 mM Tris–HCl, 150 mM NaCl, and 0.1% (v/v) Nonidet-P40, pH 7.4), the protein complexes were eluted from resin with elution buffer (30 mM Tris–HCl, 150 mM NaCl, and 4 mM biotin, pH 7.4) and further incubated with magnetic beads conjugated with mouse anti-FLAG antibody (Bimake, Shanghai, China). After incubation for 2 h at 4°C, the beads were washed with washing buffer once and TBS (30 mM Tris–HCl and 150 mM NaCl, pH 7.4) twice, followed by elution with SDT buffer (4% SDS and 100 mM Tris–HCl, pH 7.4). The tandem affinity purification was repeated two more times. One portion of the eluates was subjected to SDS-PAGE analysis (4–20% gradient gel), while another portion of eluates was digested by trypsin using the filter-aided sample preparation method, as described previously (Wisniewski et al., 2009). Briefly, dithiothreitol (DTT) was added to each sample at the final concentration of 40 mM and mixed at 600 rpm for 1.5 h at 37°C. After the samples were cooled to RT, iodoaceamide was added at the final concentration of 20 mM and incubated for 30 min in darkness to block reduced cysteine residues, followed by the transfer of samples to the filters (Microcon, 10 kDa). The filters were washed three times with 100 μL of UA buffer (8 M urea and 150 mM Tris–HCl, pH 8.0) and twice with 100 μL of 25 mM NH_4_HCO_3_ buffer. Finally, trypsin was added to the samples at the ratio of 1:50 [trypsin: protein (wt/wt)] and incubated at 37°C for 15–18 h, and the resulting peptides were collected as a filtrate. The peptides of each sample were desalted using C18 cartridges, lyophilized, and reconstituted in 40 μL of 0.1% (v/v) formic acid. The concentration of peptide was determined using UV light at 280 nm. For DIA-MS experiments, indexed retention time (iRT) calibration peptides were spiked into the samples. The peptides from each sample were analyzed using the Orbitrap™ Astral™ mass spectrometer (Thermo Scientific) connected to a Vanquish Neo system liquid chromatography (Thermo Scientific) in DIA mode. Precursor ions were scanned at a mass range of 380–980 m/z, MS1 resolution: 240000 at 200 m/z, normalized AGC target: 500%, and maximum IT: 5 ms. A total of 299 windows were set for DIA mode in MS2 scanning at isolation window: 2 m/z, HCD collision energy: 25 ev, normalized AGC target: 500%, and maximum IT: 3 ms. DIA data were analyzed using DIA-NN 1.8.1. Spectra were searched against the UniProt database (human reference proteome, January 2023 version). Main software parameters were set as follows: enzyme: trypsin, maximum missed cleavages: 1, fixed modification: carbamidomethyl (C), and dynamic modification: oxidation (M) and acetyl (protein N-term). All reported data were based on 99% confidence for protein identification as determined by a false discovery rate (FDR) ≤ 1%.

The mass spectrometry proteomics data have been deposited in the ProteomeXchange Consortium^1^ via the iProX partner repository with the dataset identifier PXD057377.

### 2.2 Co-immunoprecipitation and Western blot analysis

The co-immunoprecipitation assay was performed as described in a previous study (Tang et al., 2023). Briefly, AFAP-SF-expressing HEK293 cells were lysed in 1 mL of immunoprecipitation buffer (IP) (30 mM Tris–HCl, 150 mM NaCl, and 0.5% (v/v) Nonidet-P40, pH 7.4), supplemented with protease inhibitor cocktail (APExBIO). After cell lysate was cleared by centrifugation at 16,000 *g* for 10 min at 4°C, the supernatant was subjected to co-immunoprecipitation in a tube containing 2 μg rabbit polyclonal anti-FLAG antibody (cat#: AP0007, Bioworld Technology, MN, USA), rabbit polyclonal anti-nucleophosmin (NPM1) antibody (cat#: 10306-1-AP, Proteintech, Wuhan, China), rabbit polyclonal anti-DNA damage-binding protein 1 (DDB1) antibody (cat#: BS78489, Bioworld Technology), or rabbit polyclonal anti-HA tag antibody (cat#: D110004, BBI Life Sciences, Shanghai, China). After incubation at 4°C for 2 h, 20 μL of protein A agarose (Santa Cruz Biotechnology) was added into each tube and rotated for 2 h at 4°C. Protein A agarose resin was washed four times with IP buffer and eluted by boiling for 5 min in 40 μL 2 x SDS-PAGE sample loading buffer containing 200 mM DTT. A volume of 10 μL of the precipitates was probed using Western blot analysis with mouse monoclonal anti-FLAG antibody (cat#: M20008, Abmart, Shanghai, China), mouse monoclonal anti-NPM1 antibody (cat#: sc-32256, Santa Cruz Biotechnology), and rabbit polyclonal anti-DDB1 antibody (cat#: BS78489, Bioworld Technology).

For the Western blot analysis, immunoprecipitates in reducing 2 × SDS-PAGE sample loading buffer were subjected to SDS-PAGE analysis with 8% or 10% polyacrylamide resolving gels. The proteins were transferred to nitrocellulose membranes using the Wix-miniBlot (WIX TECHNOLOGY BEIJING CO, Beijing, China), according to the manufacturer’s instructions. The membranes were probed with primary antibodies including mouse monoclonal anti-FLAG antibody (1:1000 dilution), mouse monoclonal anti-NPM1 antibody (1:1000 dilution), or rabbit anti-DDB1 antibody (1:1000 dilution) at RT for 1 h. After washing three times with 1 x PBS (10 min each time), the membranes were incubated with secondary antibody, peroxidase-conjugated goat anti-mouse IgG (1:2000 dilution) (cat#: 5220–0341, KPL, Gaithersburg, MD), or peroxidase-conjugated goat anti-rabbit IgG (1:2000 dilution) (cat#: A16096, Thermo Scientific) at RT for 1 h. The membranes were washed four times with 1 x PBS (10 min each time) and subjected to ECL chemiluminescence. The membranes were imaged using the Tanon ABL X5 chemiluminescence imaging system (Tanon, Shanghai, China). For determination of cleaved poly (ADP-ribose) polymerase (PARP) in etoposide-treated HEK293 cells, HEK293 cells were lysed in sample buffer (6 M urea, 62.5 mM Tris–HCl, 10% glycerol, 200 mM DTT, 2% SDS, and 0.00125% bromophenol blue, pH 6.8) with 15-s sonication and 15-min incubation at 65°C. After separation by SDS-PAGE using 10% polyacrylamide resolving gels, the proteins were transferred to a nitrocellulose membrane, and sequentially probed with mouse monoclonal anti-β-actin antibody (1:1000 dilution) (cat#: AF0003, Beyotime Biotechnology, Shanghai, China), followed by peroxidase-conjugated goat anti-mouse IgG. The membrane was then probed with rabbit monoclonal anti-cleaved PARP antibody (1:1000 dilution) (cat#: AF1567, Beyotime Biotechnology), followed by peroxidase-conjugated goat anti-rabbit IgG. Imaging was taken after probing with an anti-β-actin antibody and with an anti-cleaved PARP antibody, respectively. The band intensities were determined using ImageJ software.^2^

### 2.3 Transfection and confocal microscopy

To determine the nucleolar localization of AFAP, RF/6A cells were transfected with plasmid pAFAP-GFP or pEFP-N1 using Lipofectamine 3000 (Thermo Scientific), according to the manufacturer’s instructions. pAFAP-GFP expresses GFP at the C-terminus of AFAP as a fusion protein, as described in a previous study (Tang et al., 2020). At 1 day post-transfection, cells on coverslips were fixed with 4% paraformaldehyde at RT for 20 min, followed by incubation with permeabilization and blocking solution (1% BSA/0.1% Triton X-100/PBS) for 1 h at RT. Permeabilized cells were incubated with a mouse monoclonal anti-nucleolin antibody (cat#:sc-17826, Santa Cruz Biotechnology) or mouse monoclonal anti-NPM1 antibody (cat#: sc-32256, Santa Cruz Biotechnology) at the dilution of 1:100 for 1 h at 37°C before incubation with Alexa Fluor 555-conjugated goat anti-mouse IgG antibody (1:200 dilution, Molecular Probes, Invitrogen). The cells were applied with a mounting medium containing DAPI (cat#: P0131, Beyotime Biotechnology) and subjected to observation under a Nikon Eclipse Ti2 confocal microscope.

### 2.4 Apoptosis induction, caspase-3 activity assay, and PARP cleavage assay

1 × 10^5^ HEK293 cells stably expressing AFAP-SF or SF were treated with 50 μM etoposide for 2.5 h at 37°C, followed by incubation with 1 μM GreenNuc Caspase-3 Substrate from the GreenNuc Caspase-3 Assay Kit for Live Cells (cat#: C1168, Beyotime Biotechnology) for 30 min at 37°C. The cells were observed under a microscope (EVOS M5000 imaging system, Invitrogen) before dissociation from the culture plate for flow cytometry (NovoCyte Quanteon Flow Cytometer, Agilent Technologies). Gating was applied in the SSC-H vs. FCS-H density plot to select the population using NovoExpress software (Agilent Technologies), which excluded the debris. The cells within the gate were further analyzed to calculate the percentage of cells with green fluorescence. For determination of cleaved PARP in apoptosis-induced HEK293 cells, the treatment of HEK293 cells stably expressing AFAP-SF or SF with etoposide was extended for 48 h, followed by the Western blot analysis using mouse monoclonal antibody against β-actin and rabbit monoclonal antibody against cleaved PARP.

### 2.5 Statistical analyses and bioinformatic analysis

Student’s *t*-tests were used to statistically compare protein abundance between AFAP-SF and control SF samples, both of which had at least two quantitative data obtained from mass spectrometry in three replicates, as well as to compare the percentage of cells with green fluorescence in the caspase-3 activity assay. A *p*-value of <0.05 is considered statistically significant.

For subcellular localization analysis, the multi-class SVM classification system CELLO^3^ was used to predict protein subcellular localization (Yu et al., 2006).

For the Gene Ontology (GO) term annotation and enrichment analysis, 586 proteins were annotated to the GO terms in three different categories such as biological process (BP), molecular function (MF), and cellular component (CC) by using Blast2GO software. The enrichment analysis was performed based on Fisher’s exact test, considering the whole quantified proteins as the background dataset. The Benjamini–Hochberg correction for multiple testings was applied to adjust derived *p*-values. Only GO terms with *p*-values under a threshold of 0.05 were considered significant.

## 3 Results

### 3.1 Proteomic analysis for AFAP-interacting proteins in host cells

**Figure 1:**
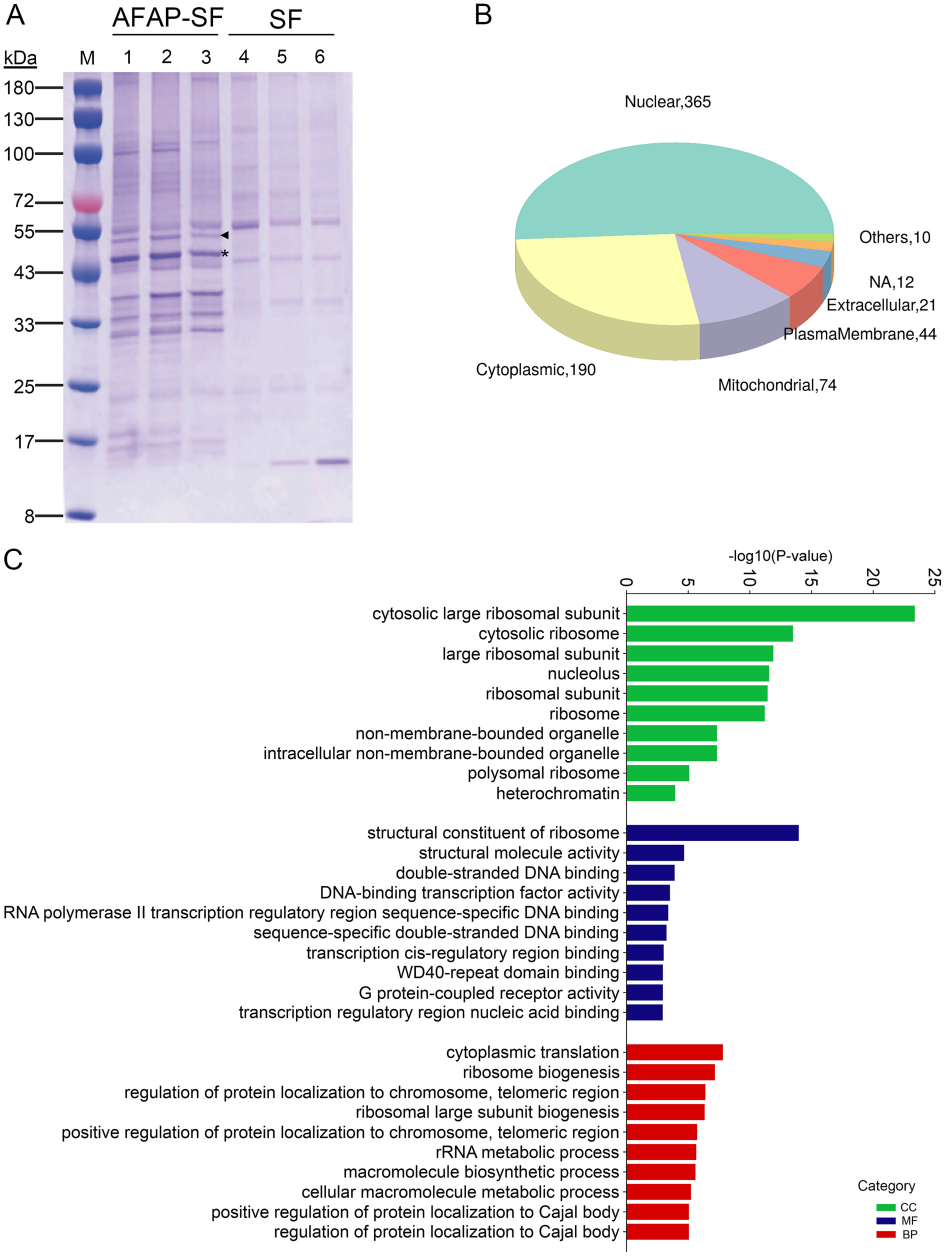
Proteomic analysis for AFAP-interacting proteins. **(A)** SDS-PAGE analysis for protein complexes isolated by tandem affinity purification from AFAP-SF- or SF-expressing HEK293 cells. Lanes 1–3 were isolated protein complexes from AFAP-SF-expressing cells. Lanes 4–6 were isolated protein complexes from SF-expressing cells. The asterisk indicates the full-length AFAP-SF band with the expected size (41.4 kDa). The triangle indicates another form of AFAP-SF band, whose size is larger than expected (approximately 50 kDa). **(B)** Subcellular localization analysis for selected 586 proteins identified in proteomic analysis. Others: lysosome, ER, Golgi apparatus. NA, not determined. **(C)** GO enrichment analysis for 586 proteins. The top 10 over-presented GO terms from each category are listed. BP, biological process; MF, molecular function; CC, cellular component.

As described previously, host nucleolin was identified as an interacting protein of AFAP (Tang et al., 2023). AFAP is an *A. phagocytophilum* T4SS effector protein that harbors four tandem repeats in its 322 amino acids (Tang et al., 2020). Nucleolin mainly localizes in the nucleolus and interacts with a multitude of proteins involved in ribosome biogenesis, gene expression, and cell growth (Salvetti et al., 2016). AFAP may extensively interact with other proteins through the interaction with nucleolin. To determine whether AFAP interacts with a multitude of proteins like nucleolin does, tandem affinity purification combined with DIA-MS was performed to identify components of purified complexes. Multiple bands with different sizes were observed in AFAP-SF lanes but were absent from control lanes (SF) in the SDS-PAGE analysis for purified complexes (Figure 1A). DIA-MS is a recently developed proteomic methodology that offers high reproducibility, accurate quantification, and broad protein coverage (Krasny and Huang, 2021). With DIA-MS, as many as 3,000 proteins were identified in AFAP-SF samples (Supplementary Table S1). Among these proteins, 214 proteins were identified in AFAP-SF samples with fold change (FC) > 3.0 in protein abundance and *p* < 0.05 (*t*-test), compared to the SF samples (Supplementary Table S2). In addition, 372 proteins were identified in two or three AFAP-SF samples but were absent from protein identification in all three SF samples (Supplementary Table S2). Thus, a total of 586 proteins were considered potential AFAP-interacting partners and used for further analyses. Subcellular localization analysis showed that the majority of these proteins (365 proteins) localize to the nucleus (Figure 1B). Functional enrichment analysis using GO terms showed that these proteins were annotated with statistical significance (*p* < 0.05) to 501 terms (Supplementary Table S3), including 360 biological process terms (BP), 68 molecular function terms (MF), and 73 cellular component terms (CC), suggesting that AFAP may be involved in diverse cellular processes. Among these terms, the top over-presented ones are related to ribosome biogenesis, ribosome, nucleolus, DNA binding, and rRNA metabolic process (Figure 1C and Supplementary Table S3). Overall, 47 proteins are ribosome subunit proteins (CC, GO:0003735), 77 proteins are annotated to ribosome biogenesis (BP, GO:0042254), 141 proteins are nucleolar (CC, GO:0005730), 102 proteins are involved in DNA binding (MF, GO:0003677), and 62 proteins participate in rRNA metabolic process (BP, GO:0016072). Table 1 shows the top 25 most abundant proteins, which include 2 abundant nucleolar proteins (nucleolin and NPM1), 17 ribosome subunit proteins, 3 chaperone proteins, 1 DNA damage repair protein (DDB1), 1 cytoskeleton protein (tubulin alpha-1C chain, TUBA1C), and 1 histone protein (Histone H1.2, H1-2), indicating that AFAP may be involved in ribosome biogenesis, DNA damage repair, cytoskeleton regulation, and nucleosome assembly.

**Table 1:** Top 25 most abundant AFAP-interacting proteins.

Protein name	FC (AFAP-SF/SF)	*p*-value
Nucleolin (NCL)	23.5	0.012
60S ribosomal protein L12 (RPL12)	3.9	0.015
60S ribosomal protein L7a (RPL7A)	11.6	0.014
60S ribosomal protein L13 (RPL13)	14.7	0.025
Heat shock 70 kDa protein 6 (HSPA6)	3.8	0.002
Nucleophosmin (NPM1)	5.5	0.012
Heat shock cognate 71 kDa protein (HSPA8)	3.9	0.002
60S ribosomal protein L10a (RPL10A)	5.8	0.003
Endoplasmic reticulum chaperone BiP (HSPA5)	7.4	0.008
DNA damage-binding protein 1 (DDB1)	13.8	0.007
60S ribosomal protein L17 (RPL17)	6.9	0.006
60S ribosomal protein L4 (RPL4)	15.3	0.008
60S acidic ribosomal protein P0 (RPLP0)	4.6	0.005
60S ribosomal protein L18 (RPL18)	16.4	0.026
60S ribosomal protein L6 (RPL6)	19.1	0.023
60S ribosomal protein L3 (RPL3)	12.1	0.003
40S ribosomal protein S3a (RPS3A)	4.4	0.004
60S ribosomal protein L27a (RPL27A)	9.2	0.012
60S ribosomal protein L10 (RPL10)	8.2	0.003
60S ribosomal protein L8 (RPL8)	7.7	0.009
60S ribosomal protein L7 (RPL7)	13.6	0.006
Tubulin alpha-1C chain (TUBA1C)	3.2	0.003
Histone H1.2 (H1-2)	10.3	0.038
60S ribosomal protein L19 (RPL19)	12.5	0.002
60S ribosomal protein L13a (RPL13A)	12.8	0.010

### 3.2 AFAP targets host nucleolus and interacts with nucleolar proteins

**Figure 2:**
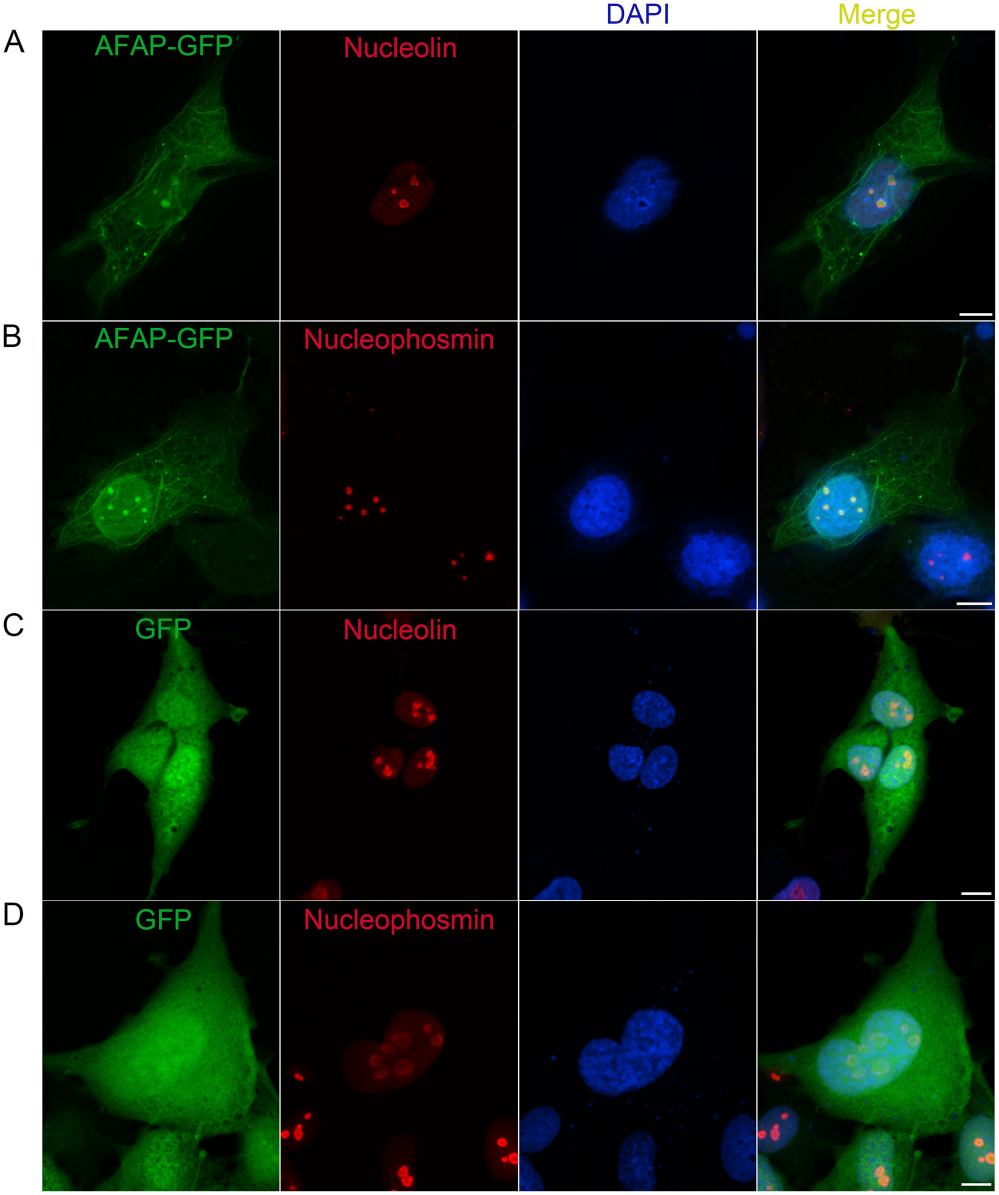
Colocalization assay for AFAP and nucleolin, and AFAP and NPM1. RF/6A cells were transfected for 1 day with a plasmid expressing AFAP-GFP **(A,B)** or GFP **(C,D)**, followed by incubation with mouse monoclonal anti-nucleolin **(A,C)** or anti-nucleophosmin **(B,D)** antibody and Alexa Fluor 555-conjugated goat anti-mouse IgG antibody. DAPI was used to stain the nucleus. Scale bars: 10 μm.

The proteomic analysis showed that AFAP interacts with nucleolar proteins. To confirm that AFAP is physically adjacent to these proteins, we performed a colocalization assay. RF/6A cells, which are flat and used for *A. phagocytophilum* propagation, were transfected with pAFAP-GFP, followed by immunolabeling with an anti-nucleolin antibody or anti-NPM1 antibody. Confocal microscopy showed that in addition to forming filament-like structures in the cytoplasm, AFAP-GFP inside the nucleus (DAPI staining) was colocalized with nucleolin and NPM1 (Figures 2A,B). Meanwhile, as negative controls, GFP was not found colocalized with nucleolin and NPM1 (Figures 2C,D), indicating that the immunolabeling was specific. These results suggested that AFAP targets the host nucleolus.

**Figure 3:**
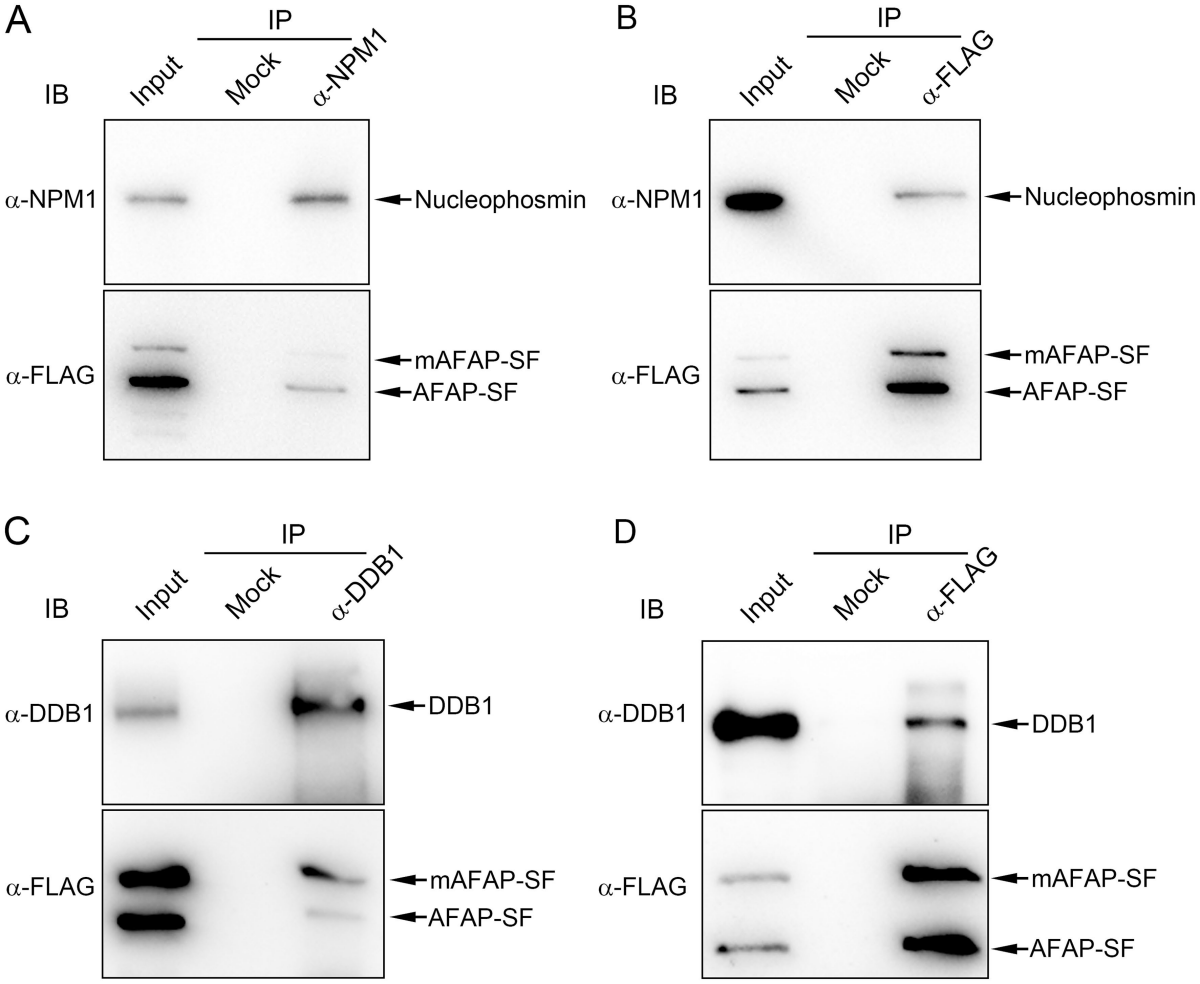
Co-immunoprecipitation assay for the interaction between AFAP and NPM1, and AFAP and DDB1. The cell lysates (input) from HEK293 cells stably expressing AFAP-SF were subjected to immunoprecipitation (IP) with rabbit polyclonal anti-HA antibody (mock), rabbit polyclonal anti-nucleophosmin antibody (*α*-NPM1) **(A)**, rabbit polyclonal anti-FLAG antibody (α-FLAG) **(B,D)**, and rabbit polyclonal anti-DDB1 antibody (α-DDB1) **(C)**. Immunoprecipitates were immunoblotted (IB) with mouse monoclonal anti-nucleophosmin (α-NPM1) antibody, mouse monoclonal anti-FLAG antibody (α-FLAG), or rabbit polyclonal anti-DDB1 antibody (α-DDB1). Of note, two bands in cell lysates and immunoprecipitates were detected by using an anti-FLAG antibody. The lower band is the full-length AFAP-SF, based on the expected size (41.1 kDa). The upper band, whose size is approximately 50 kDa, is considered a modified form of AFAP-SF (mAFAP-SF).

To validate the protein interaction in proteomic analysis, co-immunoprecipitation assays were performed to confirm the interaction of AFAP with proteins of interest. As we described in a previous study, the anti-FLAG (against AFAP-SF) and anti- nucleolin antibodies precipitated AFAP-SF and nucleolin (Tang et al., 2023). Additionally, the anti-FLAG and anti-NPM1 antibodies pulled down AFAP-SF and NPM1 (Figures 3A,B), while the anti-FLAG and anti-DDB1 antibodies pulled down AFAP-SF and DDB1 (Figures 3C,D). These results indicated that AFAP interacts with nucleolar proteins, NPM1 and DDB1, validating the result of proteomic analysis. Of note, two bands in cell lysates and immunoprecipitates were detected using the Western blot analysis with an anti-FLAG antibody. The size of the lower band meets the expected size of AFAP-SF (41.1 kDa). The upper band, whose size is approximately 50 kDa, may be a modified form of AFAP-SF (mAFAP-SF).

### 3.3 AFAP inhibits induced apoptosis

**Figure 4:**
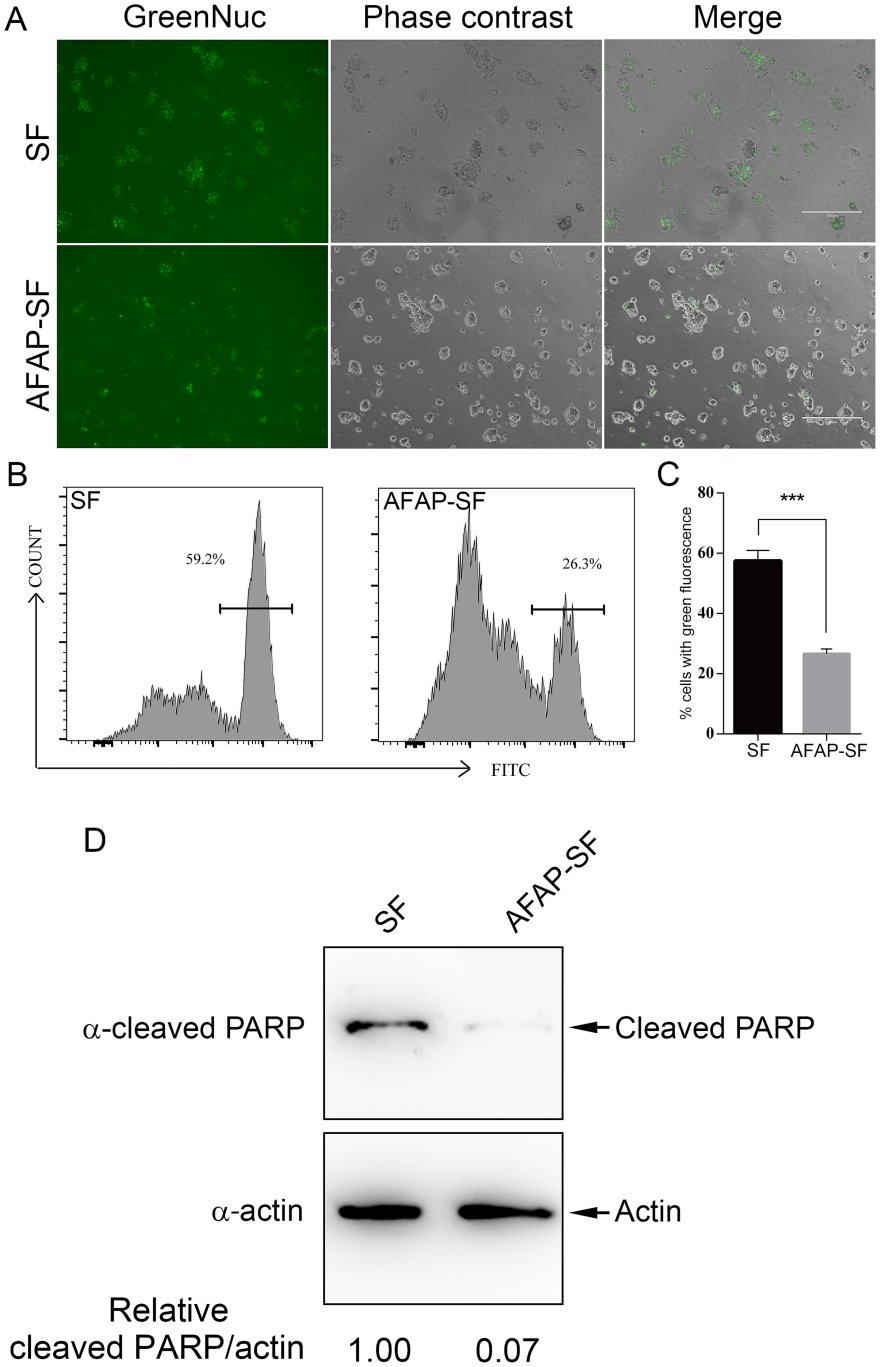
AFAP inhibits induced apoptosis. **(A)** HEK 293 cells stably expressing SF or AFAP-SF were treated with etoposide, followed by incubation with GreenNuc Caspase-3 Substrate. Cells were observed under a fluorescence microscope. Scale bars: 300 μm. **(B)** Representative flow cytometry histograms showing cell populations with green fluorescence in SF- or AFAP-SF-expressing HEK293 cells after treatment with etoposide and incubation with GreenNuc Caspase-3 Substrate. **(C)** Percentages of cells with green fluorescence after treatment with etoposide and incubation with GreenNuc Caspase-3 Substrate. SF: HEK293 cells stably expressing SF. AFAP-SF, HEK293 cells stably expressing AFAP-SF. ***Significant difference (*p* < 0.01) between groups indicated with lines using Student’s *t*-test. **(D)** HEK 293 cells stably expressing SF or AFAP-SF were treated with etoposide, followed by Western blot analysis sequentially using mouse anti-β-actin (α-actin) and rabbit anti-cleaved PARP (α-cleaved PARP) antibodies to probe the same membrane. Actin was used as an internal control to normalize the sample loading amount. Relative intensity ratios of cleaved PARP/actin bands are shown below each lane with the lane SF, set at 1.00.

In addition to the site for ribosome biogenesis, the nucleolus was recently demonstrated as the central hub in sensing and responding to cellular stress, such as DNA damage, starvation, and chemotherapeutic agents (Yang et al., 2018). When apoptotic pathways are activated in cells, NPM1 translocates from the nucleolus to nucleoplasm, where it sequesters HDM2 (human double minute 2), an E3 ubiquitin ligase, inhibiting the degradation of p53 by the proteasome, thereby leading to DNA damage response and cell arrest (Yang et al., 2018). In addition to NPM1, several ribosomal proteins, such as RPL5, RPL11, and RPL23, bind HDM2 and block HDM2-mediated p53 ubiquitination and degradation (Yang et al., 2018). AFAP targets the host nucleolus and interacts with NPM1 and RPL5, raising the potential for regulating cell stress response. Furthermore, 20 proteins annotated to the GO term, signal transduction by p53 class mediator (GO:0072331) (Supplementary Table S3), were identified in proteomic analysis, including p53, PML, RPL26, PPP2R5C, GTSE1, BAG6, SIRT1, ATAD5, USP28, NPM1, EEF1E1, RRP8, MYBBP1A, MTOR, NOP2, RPL5, BOP1, URB2, USP7, and RPL37. Additional proteins related to DNA damage response or apoptosis were also identified in this proteomic analysis, including DDB1 (Iovine et al., 2011), DCAF1 (DDB1- and CUL4-associated factor 1) (Huang et al., 2022), histone H1.2 (Lai et al., 2021), and others. Given that dozens of AFAP-interacting proteins are involved in the DNA damage response or apoptosis, we determined the effect of AFAP on the induced apoptosis. HEK293 cells stably expressing AFAP-SF or SF were treated with the apoptosis inducer etoposide, which is a topoisomerase II inhibitor, followed by incubation with GreenNuc Caspase-3 Substrate. The GreenNuc harbors a short peptide, DEVD, which is linked to the DNA-binding fluorescence dye. After loading into the cells with activated caspase-3 (an apoptosis marker), the peptide DEVD in GreenNuc is cleaved, releasing fluorescence dye, which binds nuclear DNA and makes cells fluorescent. Compared to SF-expressing HEK293 cells, fewer AFAP-SF-expressing HEK293 cells showed fluorescence under a microscope (Figure 4A). To quantify the percentage of cells with fluorescence in both groups after etoposide treatment, GreenNuc-loaded cells were subjected to flow cytometry. Compared to SF-expressing HEK293 cells, which have 57.5% fluorescent cells, 26.6% of AFAP-SF-expressing HEK293 cells show fluorescence (Figures 4B,C). Furthermore, to reinforce the results of the caspase 3 activity assay, a PARP cleavage assay was performed. During apoptosis, PARP is cleaved by activated caspases, which is considered a reliable marker of apoptosis (Oliver et al., 1998). After the treatment of etoposide, HEK239 cells expressing AFAP-SF showed 10-fold less of cleaved PARP in band density compared to HEK239 cells expressing SF (Figure 4D). These results indicated that AFAP strongly inhibits etoposide-induced apoptosis.

## 4 Discussion

In this study, 586 proteins were identified as AFAP-interacting proteins through proteomic analysis and annotated to 501 GO terms in GO enrichment analysis. The most over-represented terms were related to ribosomes, the nucleolus, DNA binding, and rRNA metabolic processes. Subcellular localization analysis showed that these proteins are mainly localized to the nucleus. Immunofluorescence labeling showed that in addition to the filament-like structures, the punctate structures formed by AFAP-GFP were localized to the host nucleoli. The nucleolus is a non-membrane-bound subnuclear body that is primarily responsible for ribosome biogenesis. As ribosomes are responsible for protein biosynthesis, an essential biological process in cells, ribosome biogenesis needs modulation responding to cell growth demand and cellular stress (Jiao et al., 2023). A total of 77 identified proteins, including 23 ribosome subunit proteins, RNA helicases, NPM1, ribosome production factor 1, ribosome biogenesis protein BOP1, rRNA processing proteins and methyltransferases, exosome complex component, and exportin-1, are related to ribosome biogenesis, according to the GO term annotation (BP, GO:0042254) (Supplementary Table S3). Ribosome biogenesis is initiated by rDNA transcription. Overall, 19 AFAP-interacting proteins are related to rDNA transcription, including UBF (upstream binding factor, a trans-acting factor mediating the recruitment of RNA polymerase I to rDNA promoter regions), as indicated by the GO term annotation for transcription by RNA polymerase I (BP, GO:0006360). rDNA transcription may be important for intracellular replication of pathogens as *L. pneumophila* effector protein LegAS4 promotes rDNA transcription (Li et al., 2013). However, the effect of AFAP on rDNA transcription and ribosome biogenesis is currently unknown and needs to be elucidated in future studies.

In addition to participating in ribosome biogenesis, ribosomal proteins, such as RPL3, RPL5, RPL6, RPL18, and RPS8, also take part in regulating multiple cellular processes including cell cycle, DNA repair, maintenance of genome integrity, apoptosis, and autophagy (Pecoraro et al., 2021). The identification of these apoptosis-related ribosomal proteins, as well as proteins annotated to the GO term, ‘signal transduction by p53 class mediator’ (BP, GO:0072331) in proteomic analysis, indicates that AFAP may play a role in regulating apoptosis. This is further supported by the caspase-3 activity assay and PARP cleavage assay in this study, which show that AFAP is capable of inhibiting induced apoptosis.

Nucleolin, identified here as the most abundant AFAP-interacting protein, promiscuously interacts with other proteins (Salvetti et al., 2016). It was found that nucleolin interacts with 144 proteins, most of which participate in ribosome biogenesis and pre-mRNA processing (Salvetti et al., 2016). Many AFAP-interacting proteins are also involved in ribosome biogenesis, suggesting that AFAP may participate in this biological process through AFAP-nucleolin interaction. However, AFAP may play additional roles in other cellular processes, as indicated by the identification of distinct binding partners, such as DDB1, DCAF1, and tubulins. Tubulins and microtubule-associated proteins were identified as AFAP-binding proteins, indicating that AFAP may specifically interact with the cytoskeleton. Indeed, filament-like structures were observed in the cytoplasm of AFAP-GFP-expressing RF/6A cells. In HeLa cells, AFAP-GFP was primarily found associated with actin filaments. However, only a few actin- or actin filament-related proteins were identified with low abundance as AFAP-interacting proteins in this study, such as microtubule-actin cross-linking factor 1 (MACF1), actin-binding protein WASF1, and formin-binding protein 4 (FNBP4). One possible reason why only a few actin- or actin filament-related proteins were identified is that the experimental conditions used in tandem affinity purification, such as low temperature, and the lack of divalent cations and ATP in cell lysis buffer, might disrupt the interaction between AFAP and actin- or actin filament-related proteins since these conditions cause disassembly of actin filaments. Another possibility is that AFAP associates with actin filament through actin filament-microtubule interactions (Dugina et al., 2016) since AFAP binds microtubule proteins. Of note, the interaction of AFAP with nucleolar proteins, such as NPM1 and DDB1, was also validated by co-immunoprecipitation assay. Meanwhile, both forms of AFAP-SF (full-length AFAP-SF and modified AFAP-SF) were found to interact with NPM1 and DDB1. In the previous study by Tang et al. (2023), only full-length AFAP-SF was detected in cell lysate and immunoprecipitates. However, two forms of AFAP-SF (full-length AFAP-SF and modified AFAP-SF) were detected in cell lysate and immunoprecipitates. The appearance of modified AFAP-SF in this study might be due to the culture condition using another source of fetal bovine serum.

The subcellular localization analysis showed that 365 AFAP-interacting proteins localize to the nucleus, and according to the GO annotation, nucleolus (CC, GO:0005730), 141 proteins are nucleolar. The other proteins may localize to other nuclear compartments. According to the GO annotation for RNA polymerase II transcription regulatory region sequence-specific DNA binding (MF, GO:0000977), 29 AFAP-interacting proteins bind to RNA polymerase II transcription regulatory region, including transcription factors (STAT1, ATF3, TF2D, BBX, and zinc finger proteins), the nucleosome-remodeling factor (RBBP4), and the histone-modifying enzyme (EZH2). This suggests that AFAP may regulate DNA transcription to mRNA. Of note, no nuclear localization signal has been identified in the AFAP sequence, and how AFAP translocates into the nucleus remains unknown.

*Anaplasma phagocytophilum* infects both mammalian cells and tick cells. During infection, *A. phagocytophilum* inhibits apoptosis and influences gene expression in both cell types (Yoshiie et al., 2000; de la Fuente et al., 2016). Multiple pathways contribute to apoptosis inhibition by *A. phagocytophilum*, including the maintenance of *bfl-1* mRNA levels, activation of the p38 MAPK pathway, and targeting of Ats-1 into mitochondria in neutrophils, as well as the upregulation of the JAK/STAT pathway in ticks (Choi et al., 2005; Ge et al., 2005; Niu et al., 2010; Ayllon et al., 2015). Meanwhile, RNA-Seq showed that more than 1,000 genes were differentially expressed in response to *A. phagocytophilum* infection (Villar et al., 2015; Dumler et al., 2018). However, little is known about the regulation of these differentially expressed genes. The finding that host nucleus-targeting AFAP interacts with proteins annotated to the GO terms, including the p53 signal transduction pathway and transcription factors for DNA transcription by RNA polymerase II, may shed light on the underlying mechanism in the regulation of host apoptosis and gene expression in response to *A. phagocytophilum* infection.

## Data Availability

The datasets presented in this study can be found in online repositories. The names of the repository/repositories and accession number(s) can be found in the article/Supplementary material.

## Footnotes

^1^ https://proteomecentral.proteomexchange.org

^2^ https://imagej.net

^3^ http://cello.life.nctu.edu.tw/
